# Catfish Glycoprotein, a Highly Powerful Safe Preservative of Minced Beef Stored at 4 °C for 15 Days

**DOI:** 10.3390/foods9081115

**Published:** 2020-08-13

**Authors:** Ali Osman, Seham Abdel-Shafi, Abdul-Raouf Al-Mohammadi, Nehal Kamal, Gamal Enan, Mahmoud Sitohy

**Affiliations:** 1 Biochemistry Department, Faculty of Agriculture, Zagazig University, 44511 Zagazig, Egypt; ali_khalil2006@yahoo.com; 2 Botany and Microbiology Department, Faculty of Science, Zagazig University, 44519 Zagazig, Egypt; hegazyseham@yahoo.com (S.A.-S.); noha.mohamed274@gmail.com (N.K.); gamalenan@ymail.com (G.E.); 3 Department of Science, King Khalid Military Academy, P.O. Box 22140, Riyadh 11495, Saudi Arabia; almohammadi26@hotmail.com

**Keywords:** antimicrobial, natural antioxidant, minced beef, shelf life, catfish glycoprotein, storage stability

## Abstract

Minced beef is a very perishable food product, due to its vulnerability to microbial contamination and its fast quality deterioration. In the current study, the biological efficiency of different concentrations (0, 50 and 100 µg g^−1^) of the antibacterial catfish glycoprotein (CFG) was estimated as a possible improver of the storability and safety of minced beef preserved at 4 °C for 15 days. CFG (50 and 100 µg g^−1^) could efficiently control the changes in meat pH during 15 days storage at 4 °C to be within the normal, acceptable levels (6.4 and 6.2, respectively), equalizing the level of the control for minced beef after 6 days of storage under similar conditions. Likewise, the level of metmyoglobin in minced beef stored at the same conditions was maintained at 53.67 and 46.67% by CFG supplementation at 50 and 100 µg g^−1^, respectively, at the 15th day of storage, which is comparable to the 6th day in case of the control samples. However, the antioxidant effect of CFG against lipid peroxidation was less effective. The antibacterial action of CFG was most pronouncedly powerful and efficient. Supplementation of minced beef with CFG at 50 and 100 µg g^−1^ significantly (*p* < 0.05) decreased the bacterial counts at all the time inspection points as compared to the control. After 15 days of storage, the total viable bacteria, psychrotrophic bacterial count and coliforms count were reduced to 3.12, 2.65 and 0.0 log CFU g^−1^, respectively, in response to CFG (50 µg g^−1^), and 2.41, 2.04 and 0.0 log CFU g^−1^, respectively, in response to CFG (100 µg g^−1^); this compared to 5.13, 4.78 and 2.5 in the control samples after only six days cold storage. Using CFG at 50, 100 and 200 µg g^−1^ in rat diets did not affect their liver or kidney functions, reflecting the non-toxicity of this substance. Substantiating the antioxidant and antimicrobial potential of CFG in minced beef storage may support its use as a naturally powerful and safe food preservative, as well as a shelf-life extender.

## 1. Introduction

Consumer demand for meat products has rapidly increased over the last decade. Being rich in different nutrients, meats are usually vulnerable to microbial contamination and lipid oxidation, negatively affecting public health, [1]. Increased microbial activity and enhanced lipid oxidation adversely impact the safety, nutritional value, organoleptic qualities, and economic value of the meat during storage [2]. Minced beef is a widely consumed food product that has a particularly short shelf-life. The changes in lipid and protein oxidation have been indicated as major factors decreasing the quality and shelf-life of meats [3], particularly minced meats due to their increased exposure to air and contamination [4,5]. Due to their high water and protein contents, meat products are optimal habitats for microbial growth. Microorganisms inhabiting meat are classified into two main categories: spoilage and pathogenic bacteria. The first category includes the genera *Pseudomonas*, *Lactobacillus* and *Enterococcus*, and the second one comprises *Salmonella enterica*, *S. typhimurium*, *Campylobacter jejuni*, *Escherichia coli*, *Staphylococcus aureus* and *Listeria monocytogenes* [6]. There are different strategies to ensure the safety and quality of meat products, such as high pressure, super-chilling and chemical preservatives or engineered nanoparticles [7]. Several synthetic antioxidants and antimicrobials have been utilized to hinder oxidative reactions, inhibit microbial growth and consequently extend the shelf life of minced meat products [8]. The growing consciousness of human health and the justified fear of synthetic compounds have created a research need to explore the utility of natural antimicrobials and antioxidants in extending the shelf-life of stored meat products while maintaining their quality and safety [5,9]. Various natural bio-sources, e.g., plant basic proteins [10,11], phycocyanin [12,13,14], milk proteins [15,16,17,18], herbs [19,20,21,22,23,24,25,26,27,28], chemically modified proteins [29,30,31,32,33], probiotic bacteria as well as their produced bacteriocins [34,35] may provide potential natural preservatives.

Glycoproteins represent a significant class of natural products currently reported as playing vital roles as an antimicrobial agents [36,37,38]. In a previous study, catfish glycoprotein (CFG) was isolated from the mucous obtained from the cutaneous surface of catfish growing in freshwater in Egypt. It was biochemically characterized [39] as having a single 22 KD subunit and a pH 8 isoelectric point (PI). It also exhibits a considerable antibacterial activity, i.e., low MIC (50 µg mL^−1^) against nine pathogenic bacteria (six Gram+ and three Gram−). Based on these results, the current study was an endeavor to exploit this high and wide spectrum antibacterial activity in persevering minced beef. To assure the safe use of CFG in preserving minced beef, a particular focus was put on the potential toxicity that may be associated with this protein coming from fish subjected traditionally to pollution. The current work is evaluating the antibacterial activity of CFG in situ in minced beef preserved under cold conditions for 15 days. The potential toxicity of CFG was primarily studied in Wister albino rats.

## 2. Materials and Methods

### 2.1. Catfish Glycoprotein (CFG) Isolation and Characterization

Alive fresh catfish were purchased from the local market of Zagazig, Egypt, and maintained in running water tanks to minimize bacterial contamination and to enhance the rate of mucus production. The mucus layer was carefully scraped off the catfish dorsal body surface with a plastic spatula and was immediately frozen, lyophilized and stored at 4 °C [39]. Catfish glycoprotein (CFG) was isolated from the mucus material by ammonium sulfate precipitation (55%) and purified on Sephadex G-100 according to the procedure of [18].

CFG was SDS-PAGE electrophoresed following [40]. An amount of CFG (10 mg) was dispersed in 500 µL SDS 10% and mixed with 100 µL β-mercaptoethanol for 15 min by vortexing every 5 min. The extract was centrifuged at 10,000× *g* for 10 min. A 20 µL extract of the mixture, 20 µL of SDS-loading sample buffer (SDS 4%, β-mercaptoethanol 3%, glycerol 20%, Tris HCl 50mM pH 6.8 and bromophenol blue traces), was prepared and heated at 96 °C for 3 min. An aliquot (10 μL) of the mixture was electrophoresed [41].

Measuring the CFG antioxidant activity was based on its ability to scavenge DPPH radicals according to [42]. Briefly, an aliquot (500 µL) from each CFG concentration (100, 200, 400, 800 and 1600 µg mL^−1^) was combined with a 2500 µL DPPH ethanol solution (0.1 mM) and incubated for 30 min. The optical density of the mixture was measured at 520 nm using a spectrophotometer (JENWAY, 6405 UV/Vis, and U.K.). The antioxidant activity % of the free radical DPPH was calculated as follows:

(1) $$Antioxidant\;activity\;\left(\%\;inhibition\right)=\;\left[\left(Absorbance\;of\;control-Absorbance\;of\;sample\right)/Absorbance\;of\;control\right]\times 100$$

The DPPH radical scavenging activity was calculated as the SC_50%_ value (sample concentration required to scavenge 50% of DPPH radicals) based on the decrease of the DPPH solution absorbance.

### 2.2. Preparation and Storage of Minced Beef

The fresh raw beef (protein 21%, fat 15%, ash 1% and 61% moisture), purchased from a local market in Zagazig City, Egypt, was finely minced and divided into nine portions weighing 1 kg each. These portions were randomly allocated into three equal groups, each having three replicates. The three replicates of the first group did not receive any supplementation and served as the control. For the second and third groups, each replicate was combined with 50 or 100 mg of CFG (equivalent 50 and 100 µg g^−1^ beef) and re-minced for 3 min at room temperature for homogenization. Every replicate of the minced beef samples (1 kg) was divided into 20 small samples and kept in small sterilized polyethylene sachets (50 g each). Samples were withdrawn for physicochemical and microbiological analysis at different periods (after 0, 3, 6, 9, 12 and 15 days). The control was prepared similarly but without adding CFG. All sample sachets were wrapped and stored under aerobic conditions at 4 °C for 15 days.

#### 2.2.1. Analysis of the Minced Beef Samples

The stored minced beef samples were withdrawn after 0, 3, 6, 9, 12 and 15 days of storage and transferred into stomacher bags and homogenized in a Stomacher Lab-Blender 400 (SEWARD MEDICAL, London, UK) for 2 min before taking the samples for the different analyses.

##### pH Value

Initially, a 10 g sample was combined with 50 mL of cold (4 °C) distilled water at pH 7 for 1 min. The pH was measured potentiometrically at room temperature with a microprocessor pH meter (pH 211 Hanna Instruments Inc. Nusfalau, Romania).

##### Metmyoglobin (MetMb)

The MetMb content in the minced beef was assayed by homogenizing a 10 g sample in 50 mL ice-cold 40 mM phosphate buffer (pH 6.8) for 10 s [43]. The homogenate was left for 1 h at 4 °C before centrifugation at 4500× *g* for 30 min at 4 °C. The supernatant was filtered through Whatman filter paper No.1 and the filtrate absorbance was measured at 572, 565, 545 and 525 nm using a JENWAY 6405 UV/visible spectrophotometer (UK)**.** Based on the recorded absorbance values, the MetMb content was calculated according to the following formula [44]:

(2) $$MetMb\;\left(\%\right)=\left[-2.51\left(\frac{A572}{A525}\right)+0.777\left(\frac{A565}{A525}\right)+0.8\left(\frac{A545}{A525}\right)+1.098\right]\times 100$$

##### Oxidation Stability Assay

Lipid peroxidation was evaluated in the minced beef samples following [45,46]. Five grams of each beef sample was homogenized. A total of 10 mL of the homogenate was mixed with 90 mL of 0.05 Mol/L phosphate buffer (pH 7) and centrifuged at 12,000× *g* for 60 min at 4 °C. An aliquot (100 µL) of the supernatant was combined with 2000 µL of a TBA–TCA–HCl (1:1:1) reagent (thiobarbituric acid 0.37%, 15% trichloroacetic acid and 0.25 N HCl) and placed for 30 min in a boiling water bath, and then allowed to cool. The optical density of the emergent color was recorded at 535 nm using a JENWAY 6405 UV/visible spectrophotometer (UK) against a reagent blank. The lipid oxidation inhibition % was calculated according to the following equation:

(3) $$\mathrm{Lipid}\;\mathrm{oxidation}\;\mathrm{inhibition}\;\left(\%\right)=\left[1-\left(\mathrm{absorbance}\;\mathrm{of}\;\mathrm{sample}/\mathrm{absorbance}\;\mathrm{of}\;\mathrm{control}\right)\right]\times 100$$

##### Microbial Analysis

Microbial analysis was conducted in the stored minced beef samples [47]. The samples (10 g) were aseptically transferred to a stomacher bag containing 90 mL of peptone saline diluent (1.0 g peptone and 8.5 g sodium chloride in 1 L of distilled water) and homogenized for 60 s. A serial 10-fold dilution series was prepared. Different bacterial counts were determined using different specific selective media [48,49]. The total viable count (TVC) was assessed on Plate Count Agar (PCA, Merck, Darmstadt, Germany) at 25 °C after 72 h. Psychrotrophs were assayed on PCA (Merck, Darmstadt, Germany) at 7 °C after 10 days. Coliform bacteria were determined on MacConkey agar (Mast Group, Merseyside, UK) with a double layer of the same medium incubated at 37 °C for 24 h. Microbiological data were expressed as the logarithms of the number of colony forming units (CFU g^−1^).

##### Sensory Evaluation

Sensorial evaluation of the raw minced beef treated with CFG (50 and 100 µg g^−1^) was carried out by twenty trained panelists selected from graduate students. Each panelist performed different assays (color, odor, appearance and overall acceptability) for each sample, compared to the control samples at each storage period; 0, 3, 6, 9, 12 and 15 days at 4 °C. Randomly coded samples were presented in individual booths to each panelist for evaluation. The panelists scored the sensorial qualities using a 1–9 scale (9 = like extremely; 8 = like very much; 7 = like moderately; 6 = like slightly; 5 = neither like nor dislike; 4 = dislike slightly; 3 = dislike moderately; 2 = dislike very much; and 1 = dislike extremely). Samples with scores below 5 were deemed unacceptable.

### 2.3. Analysis of CFG Safety in Wistar Albino Rats

Healthy male white albino rats (*Rattus norvegicus*), the Wistar strain (150 ± 10 g, body weight), were purchased from the Organization of Biological Products and Vaccines (Helwan farm, Cairo, Egypt) and housed in plastic cages in random groups of 8 animals/cage. The experimental animals were acclimatized to the laboratory conditions (temperature of 25 ± 5 °C; relative humidity 50–70% and a normal light/dark cycle) for two weeks before the experiment period. They were provided a balanced pelleted diet (23% protein) and tap water ad libitum throughout the whole adaptation and experimental periods. The CFG safety was tested as incorporated in the diets at three levels, namely, a 50, 100 and 200 µg g^−1^ diet compared to the control (0 µg g^−1^). Thirty-two Wistar male rats were divided into four groups of 8 rats each. Each rat received, on average, a daily diet of 100 g/kg rat weight. So, the four groups received a daily meal of a standard balanced diet containing 0, 5, 10 and 20 mg/kg body weight for 28 days. The last treatment was double the highest level of CFG used in preserving the minced beef to assure its safety [50].

At the end of the experiment (the 28th day), blood samples were collected into clean, dry and labeled Eppendorf tubes containing heparin as anticoagulant (7.5 I.U.) for the clinico-biochemical assays. Serum samples were separated from blood samples according to [51], and then stored at −20 °C until analysis. The serum content of urea, creatinine, as well as the enzymatic activities of alanine aminotransferase (ALT), aspartate aminotransferase (AST) and alkaline phosphatase (ALP) were determined using commercial diagnostic kits provided by the Bio Diagnostic Co. (Giza, Egypt).

### 2.4. Statistical Analysis

All data were subjected to statistical analysis by a one-way ANOVA test employing SPSS software. A probability of *p* < 0.05 was set as the level of significance unless stated differently.

## 3. Results

### 3.1. CFG Characterization

**Figure 1:**
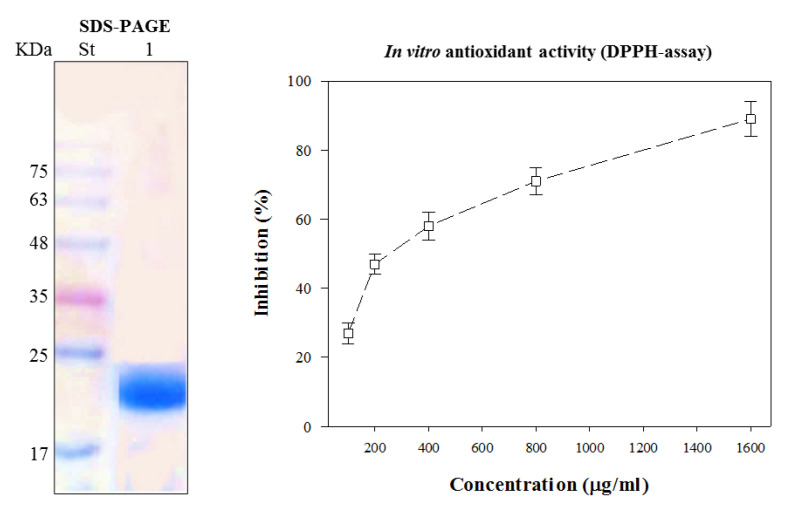
SDS-PAGE electrophoretic pattern of the purified catfish glycoprotein (Lane 1) compared to the standard molecular weight (St) and its antioxidant activity at different concentrations (100, 200, 400, 800 and 1600 µg mL^−1^), as measured by a DPPH assay. Every value is the average of three replicates ± SD.

CFG was purified on Sephadex G-100 and separated on SDS-PAGE, giving a single protein band corresponding to 22 KD (Figure 1). The iso-eclectic point of this protein was previously reported to be IEP 8. The antioxidant activity of the CFG was measured at various concentrations (0, 100, 200, 400, 800 and 1600 µg mL^−1^) in ethanol extract by the DPPH radical scavenging activity. The data presented in Figure 1 show that the radical scavenging effect increased with increasing CFG concentrations. The highest DPPH radical scavenging activity of the CFG (90% ± 2.8) was obtained at 1600 µg mL^−1^, recording SC_50%_ at 360 µg mL^−1^.

### 3.2. Storage of Minced Beef

**Figure 2:**
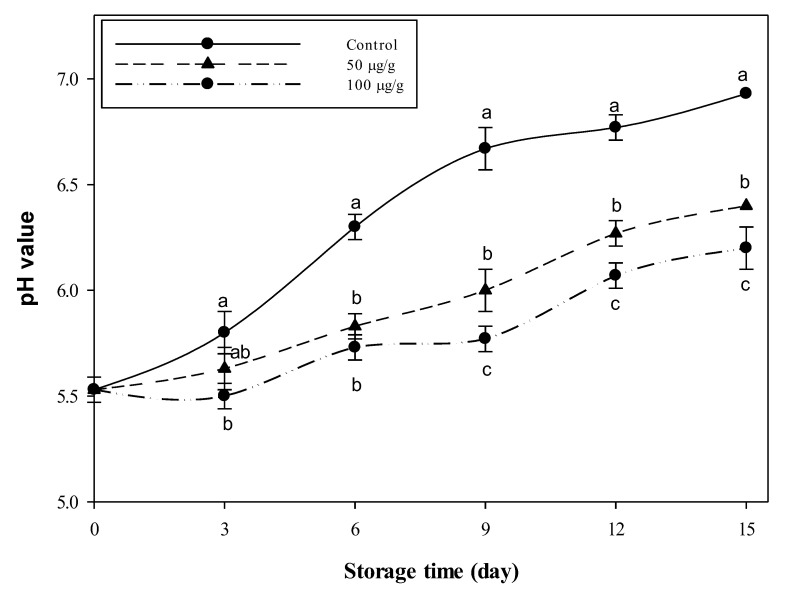
Variations in the pH of minced beef combined with catfish glycoprotein (CFG) at different levels (0, 50 and 100 µg g^−1^) during 15 days storage at 4 °C. Every value is the average of three replicates ± SD. Different letters for the same time storage refer to significant differences (*p* < 0.05).

The pH variations in the minced beef (protein 21%, fat 15%, ash 1% and 61% moisture) stored for 15 days at 4 °C, as combined with the 50 and 100 µg CFG/g meat, are presented in Figure 2. It is observed that the meat pH increased alongside the preservation time in the control and treated samples. However, the pH values of the treated samples were lower than the control at all the time points. For the first six days of storage, there were no significant differences (*p* < 0.05) between the two concentrations viz. 50 and 100 µg g^−1^ CFG. At the time points, 9–15 days, the higher concentration of CFG was significantly (*p* < 0.05) more effective than the lower one. After two weeks of storage at 4 °C, the pH of the control sample increased, reaching 6.93 while it was still 6.4 and 6.2 in the meat sample treated with CFG at 50 and 100 µg g^−1^, respectively. This pH value is equivalent to the control around the 6th day. So, it could be envisaged that chemical changes in the CFG-treated samples are highly slowed down, such that the 15-day-old meat may be equivalent to the 6th-day meat of the control sample. So, the CFG has delayed the aging process of the stored meat by about 7 days during the whole storage period.

**Figure 3:**
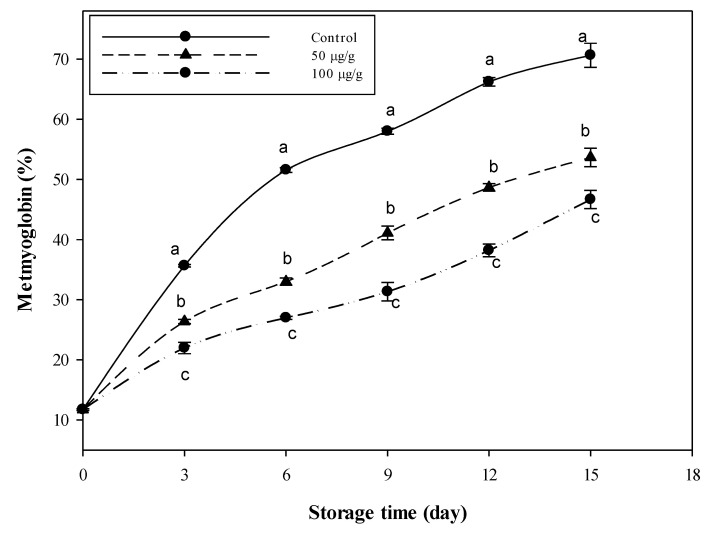
Variations in metmyoglobin (%) of minced beef combined with catfish glycoprotein (CFG) at different levels (0, 50 and 100 µg g^−1^) during 15 days storage at 4 °C. Every value is the average of three replicates ± SD. Different letters for the same time storage refer to significant differences (*p* < 0.05).

The data in Figure 3 reflect the changes in metmyoglobin, the oxidized form of the oxygen-carrying hemeprotein myoglobin. The level (%) of metmyoglobin in the untreated sample rose from 52% (after 6 days) to about 71% after 15 days of storage. The minced meat samples combined with CFG at 50 and 100 µg g^−1^ recorded lower values; only 54 and 47% after 15 days, respectively. These two values are approximately equivalent to the 7th and 5th days in the control samples. So, the sample treated with the high CFG level may delay the oxidation process by about ten days compared to the control.

**Figure 4:**
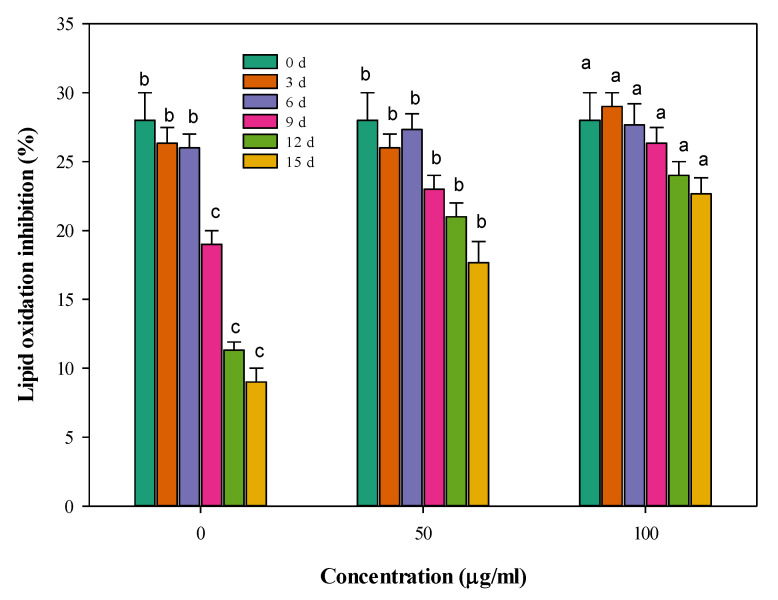
Changes in lipid oxidation inhibition (%) of minced beef combined with catfish glycoprotein (CFG) at different levels (0, 50 and 100 µg g^−1^) during 15 days storage at 4 °C. Every value is the average of three replicates ± SD. Different letters for the same time storage (same column color) refer to significant differences (*p* < 0.05).

The data in Figure 4 depict the time changes in the ability of the stored meat to inhibit lipid oxidation. It can be generally noticed that lipid oxidation increased with the storage time, i.e., the inhibition of oxidation is decreasing. In the control samples, oxidation was highly accelerated. So, the inhibition to this oxidation process was only 9% after 15 days of storage at 4 °C in the control sample. The oxidation process was significantly (*p* < 0.05) inhibited in the stored meat samples by incorporating CFG at 50 and 100 µg g^−1^. The differences between the influence of the two concentrations and the control were small until the 6th day of storage but increased with storage time. The extent of the lipid oxidation inhibition was least deteriorated when adding 100 µg g^−1^ CFG to the stored meat, where the value of the lipid oxidation process at 15 days of storage is probably equivalent to the 7th day in the control meat.

The microbiological analysis of the stored minced beef samples showed considerable increases in the total viable bacteria, psychrotrophic bacterial count and coliform count. They reached maximums of about 8.67, 7.62 and 4.45 log CFU g^−1^ after 15 days of storage at 4 °C (Table 1). Supplementation of minced beef with CFG at 50 and 100 µg g^−1^ significantly (*p* < 0.05) reduced the bacterial counts at all time inspection points compared to the control. The higher CFG concentration was always more effective than the lower one. After 15 days of storage, the three counts mentioned above were reduced to 3.12, 2.65 and 0.0 log CFU g^−1^ in response to the CFG (50 µg g^−1^) and 2.41, 2.04 and 0.0 log CFU g^−1^ in response to the CFG (100 µg g^−1^).

**Figure 5:**
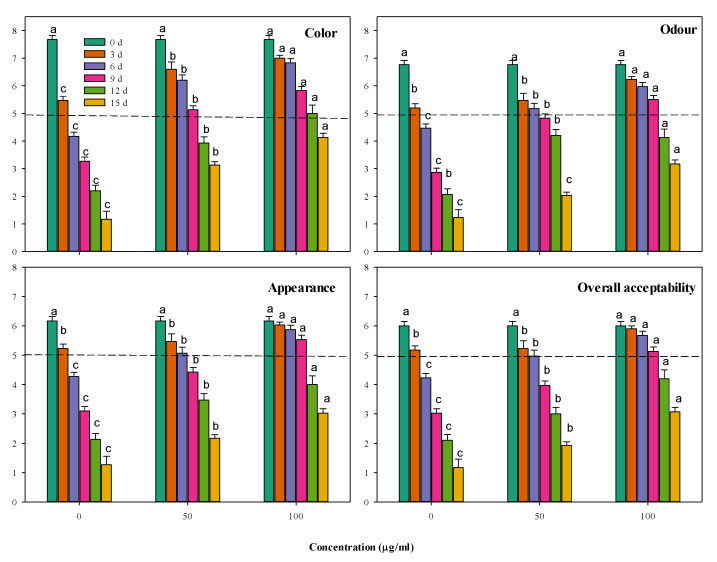
Storage changes in the sensorial properties of minced beef combined with catfish glycoprotein (CFG) at different levels (0, 50 and 100 µg g^−1^) during 15 days storage at 4 °C. Every value is the average of ten replicates ± SD. The dashed line represents the border under which the sensorial quality is considered unacceptable. Different letters for the same time storage (same column color) refer to significant differences (*p* < 0.05).

The stored meat’s sensorial traits were evaluated every three days for color, odor, appearance and overall acceptability (Figure 5). It is noticeable that the color and odor of all the minced beef samples were deteriorated by the storage. Supplementing minced beef with CFG at 50 and 100 µg g^−1^ restricted the deterioration rate. The higher concentration of CFG (100 µg g^−1^) was generally more effective than the lower one and significantly (*p* < 0.05) excelled the control by more than double after 12–15 days of storage for both the color and odor traits. These changes were reflected in the general appearance and global acceptability of the evaluated minced beef. So, supplementing the minced beef assured reasonable sensorial properties for a longer time during storage.

### 3.3. CFG Safety

The safety of CFG was tested as incorporated into the animal diet at three levels, namely, a 50, 100 and 200 µg g^−1^ diet. Each rat received an average daily intake of 100 g/kg rat weight. The four groups received daily meals of standard diets containing 0, 5, 10 and 20 mg/kg rat body weight for 28 days. The first two CFG levels (5 and 10 mg/kg rat body) are equivalent to the two levels used in meat preservation (50 and 100 µg g^−1^). The highest CFG is double the higher level used in meat preservation. It is observed in Table 2 that administering CFG-supplemented minced beef for 28 days did not significantly (*p* < 0.05) affect the levels of the liver or kidney bioindicators, which all remained within the typical values. So, using CFG in preserving minced beef at 50 and 100 µg g^−1^ is not going to affect liver or kidney functions. Even when this ratio of CFG supplementation was doubled (200 µg g^−1^), no toxicity was observed.

## 4. Discussion

The SDS-PAGE of the prepared sample showing one band corresponding to 22 KD agreed with our previous preparation [39], indicating the result’s reproducibility since the starting material was from different source samples. This result may also refer to the fact that CFG is a small-sized protein with a high iso-electric point (8 IEP), which may enable it to have high antibacterial activity, as previously substantiated [39]. The antioxidant activity of CFG measured by the DPPH radical scavenging activity showing a proportional increase with the concentration may confirm the causal relationship between the substances (CFG) and the antioxidant activity. The indicative values of CFG (1600 µg mL^−1^) achieving the highest DPPH radical scavenging activity (90% ± 2.8) and the relatively low SC_50_ of the CFG (360 µg mL^−1^), delivering a 50% inhibition, may indicate some effective antioxidant activity of the substance, following [52]. This result may, in turn, predict an impacting role in the preservation process through time. Free radical scavenging may preclude the termination of lipid oxidation, as their formation is the first step of lipid oxidation. This activity will subsequently hinder the formation of lipid off-flavors and promote product acceptability and sensorial quality, extending the shelf-life [53].

The changes in the content of metmyoglobin (%) prove that treating minced meat samples with a high level of CFG delayed the rate of the metmyoglobin oxidation process by about ten days compared to the control. So, CFG could have efficient antioxidant activity against the oxidation of myoglobin to metmyoglobin. Minced beef protected with CFG at 100 µg g^−1^ recorded a value of metmyoglobin at the 15th day of storage lower than the value associated with the control samples’ 6th day. This action is probably due to the antioxidant capacity of CFG, as substantiated by the in vitro measurements.

The results of the lipid oxidation levels during the minced beef’s storage may be referring to the slowing down of lipid peroxidation when treating minced meat with CFG, particularly at the high CFG level (100 µg g^−1^ CFG). However, this antioxidant effect against lipid peroxidation seems much lower than against myoglobin oxidation. The lipid peroxidation inhibition in the samples enriched with a high CFG level (100 µg g^−1^ CFG) could only down the process by three days; i.e., the level of lipid oxidation inhibition of 26.33 ± 1.53 at the 9th day was equivalent to the level of the control sample at the 6th day. This might imply that the antioxidant activity of CFG might have delayed the oxidative aging by about three days, which is much lower than in the case with metmyoglobin oxidation. Both of these two delayed oxidation processes are evidently due to the antioxidant activity of CFG, as evidenced by the in vitro high DPPH radical scavenging activity (90% ± 2.8) of the CFG. This lower action on inhibiting lipid rather than myoglobin oxidation may be due to some miscibility differences of the substance (CFG) with either of the protein or lipid material. Miscibility is an important factor controlling biomolecules reactivity [54,55]. Hence, to protect stored meat against lipid oxidation, some other compatible substances may be additionally added.

The microbiological parameters of the minced beef combined with the CFG at different levels (0, 50 and 100 µg g^−1^) showed various signs of amelioration. The increased pH values in the untreated meat samples with storage time may be reflecting their possible liability to microbial contamination, or even the regular biochemical changes are going to be at their maximum levels. The increasing upward trend in pH values of the control samples may be probably due to the formation of the basic microbial metabolites derived from the deamination of beef proteins [56].

Conversely, the rate of pH increase in the CFG-supplemented minced beef samples was relatively slower, in that the highest level in pH after 15 days was only 6.4 ± 0.0 and 6.2 ± 0.10 in the samples supplemented with 50 and 100 µg g^−1^ CFG, which is nearly equivalent to the control samples around the 6th day. It can be inferred that the treatments have slowed down the aging process by more than eight days. Based on this result, it can be extrapolated that the spoilage point can be moved to a time point farther than 15 days on the storage time scale, especially when supplemented by a high dose of CFG. Resisting the increase in pH may mean reducing the contamination by the bacteria producing the basic substances (e.g., ammonia, amines) and restricting protein degradation [20,57]. The pH-reducing effect of CFG on minced beef at 100 µg g^−1^ is probably equivalent to the action of legumin and nisin at a higher concentration (200 µg g^−1^), as reported in [20]. So, the current material is probably more useful than both legumin and nisin. Additionally, the pH maintaining action of CFG (100 µg g^−1^) on the preserved meat seems more effective than other natural preservatives, e.g., chickpea legumin, *Phaseolus vulgaris* protein hydrolysates, *Nigella sativa* oil and clove oil, which maintained the meat pH at around 6.9 after 14 days of cold storage [20,21,22,23], against a pH of only 6.2 in the current study.

Microbiological analysis demonstrated the highly distinct antibacterial action of CFG on the content of the three main bacterial counts in stored minced beef. The levels of the bacterial counts in low CFG (50 µg g^−1^) supplemented minced beef are comparable to the period 0–2 days of storage in the control samples. So, it can be concluded that the antibacterial action was too high to allow any significant bacterial growth during the whole period of storage. Moreover, the high concentration of CFG (100 µg g^−1^) was too powerful to allow any increase in the bacterial counts recoded at day zero of minced beef storage. These antibacterial actions are similar but higher than similar ones induced by the cationic native proteins, e.g., soy glycinin, which could limit the bacterial counts in either pasteurized or raw milk preserved under cold conditions [19,58]. It is also higher than the cationic esterified legume protein’s preservative action on pasteurized or raw milk [53,59,60,61,62,63].

This powerful antibacterial potency is extraordinary and so much distinct from other natural antibacterial proteins. For example, chickpea legumin (200 µg g^−1^) could only maintain the TVC in the preserved minced meat to 6.3 log CFU g^−1^ after 15 days under similar conditions [20] against 3.12 and 2.41 and log CFU g^−1^ by CFG (50 and 100 µg g^−1^, respectively) at the same time point. This action is also much higher than that of nisin, which could only reduce the TVC to 6.7 log CFU g^−1^ after 12 days of storage under similar conditions [20]. The distinct antibacterial action of CFG is particularly evident against the coliform count. It brings it down to the 0.0 level after 15 days cold storage despite the coliforms’ ability to proliferate during storage, both in ordinary and modified packaging conditions [64].

In conclusion, it can be stated that the tested CFG is a highly powerful preservative, and that the CFG-supplemented minced meat is quite recommendable for human consumption after more than 15 days of storage at 4 °C. However, the overall acceptability and CFG safety should be considered when deciding the most appropriate shelf-life for the supplemented product. The initial success of using this natural product (CFG) in the preservation of minced beef concluded in the current work is highly generative.

The sensorial acceptability of the stored minced beef depends on the different previous interfering factors, including the microbiological and the biochemical status of the product and their reflection on various sensorial factors, such as color, odor and appearance. It is observed that the usual storage deteriorations in color, odor and appearance were restricted by supplementing the minced beef with CFG at 50 and 100 µg g^−1^, particularly the high concentration, which produced a better product than the control. However, the influence of CFG supplementation on the sensorial qualities was not enough to maintain full product acceptability for the 15 days of cold storage. Keeping about 50% of the maximal acceptability can be witnessed after 9–10 days of storage in the samples supplanted by 100 µg g^−1^ CFG against only 3–4 days for the control samples. The failure of CFG to maintain high sensorial acceptability is perhaps due to its low action against lipid peroxidation, which contributes to the reduced sensorial acceptability. Although supplementing the minced beef with CFG assured reasonable sensorial properties for a longer storage time, it could not maximize it to the desired high levels. To guarantee a higher acceptability of the stored minced beef, other influential antioxidant factors should be incorporated in the preserved minced beef, combined with CFG.

For further assuring the safety of minced beef supplemented with CFG, a primary biological experiment tested its safety if incorporated in the diets of Albino rats at levels equal to or higher than the levels used in preserving the minced beef, i.e., a 50, 100 and 200 µg g^−1^ diet. The results showed that the CFG levels are equivalent to those used in beef preservation. Even doubling the highest tested concentration did not have any adverse effects on the experimental animals, particularly on the liver or kidney bioindicators during 28 days of administration. So, it can be concluded that using CFG in preserving minced beef at 50 and 100 µg g^−1^ does not negatively affect liver or kidney functions. Even if the ratio of CFG supplementation doubled (200 µg g^−1^), no toxicity was observed referring to its safety and entailing a detailed toxicological study on this highly active substance.

## 5. Conclusions

Microbiological control of minced beef is one of the essential approaches in promoting its quality, extending its shelf life, ensuring its safety and restricting the potential wastes. Based on the microbiological analysis results, the addition of CFG to minced beef significantly proved to be a highly powerful tool in reducing the total viable bacteria, psychrotrophic bacterial count and coliforms to minimal levels during 15 days of cold storage. Considering the obtained values, the minced beef can probably be stored for a longer period of time without major microbiological deterioration. Besides, the results showed that adding CFG could effectively restrict the changes in the pH of the minced beef to acceptable levels after 15 days of cold storage. However, the oxidative deterioration in the preserved minced beef and the incapability of CFG to efficiently alleviate it need further experimental approaches to include other antioxidant factors combined with CFG. The antioxidant effect against lipid peroxidation is much lower than against myoglobin oxidation. The lipid peroxidation inhibition in the samples enriched with a high CFG level (100 µg g^−1^ CFG) could only be slowed down by three days. This is a much lower rate than in the case of inhibiting metmyoglobin oxidation, probably due to the immiscibility factor between CFG and the meat lipid. Hence, to enhance the protection of stored minced beef against lipid oxidation, some other compatible substances or more powerful antioxidants may be needed.

Using CFG in preserving minced beef at 50 and 100 µg g^−1^ can be recommended as a food-safe treatment since adding these ratios to Wistar albino rats for 28 days did not negatively affect liver or kidney functions or trigger any toxicity signs in the animals, even when the CFG level was up to 200 µg g^−1^. The results obtained in this study support the recommendation of CFG as a safe, natural preservative for minced beef, capable of maintaining its overall quality for at least nine days and its microbial validity for more than 15 days under cold conditions.

## Figures and Tables

**Table 1:** Total viable count, psychrotrophic bacterial count and coliforms count (log CFU g^−1^) in minced beef treated with catfish glycoprotein (CFG) at different concentrations (0, 50 and 100 μg/g) and stored at 4 °C throughout the storage period of 0–15 days.

Storage Time (day)	Control	50 µg g^−1^	100 µg g^−1^
	Total Viable Count (log CFU g^−1^)		
0	3.77 ± 0.15	3.77 ± 0.15	3.77 ± 0.15
3	4.90 ± 0.10 ^a^	3.71 ± 0.06 ^b^	3.56 ± 0.05 ^c^
6	5.13 ± 0.11 ^a^	3.57 ± 0.13 ^b^	3.56 ± 0.05 ^b^
9	6.90 ± 0.10 ^a^	3.61 ± 0.02 ^b^	3.23 ± 0.05 ^c^
12	7.27 ± 0.25 ^a^	3.40 ± 0.08 ^b^	3.27 ± 0.06 ^b^
15	8.67 ± 0.32 ^a^	3.12 ± 0.11 ^b^	2.41 ± 0.11 ^c^
**Psychrotrophic Bacterial Count (log CFU g^−1^)**			
0	2.09 ± 0.05	2.09 ± 0.05	2.09 ± 0.05
3	2.82 ± 0.08 ^a^	2.09 ± 0.05 ^b^	2.03 ± 0.03 ^b^
6	4.78 ± 0.05 ^a^	2.85 ± 0.05 ^b^	2.29 ± 0.03 ^c^
9	5.79 ± 0.09 ^a^	2.93 ± 0.04 ^b^	2.50 ± 0.02 ^c^
12	6.58 ± 0.06 ^a^	2.94 ± 0.04 ^b^	2.73 ± 0.05 ^c^
15	7.62 ± 0.22 ^a^	2.65 ± 0.03 ^b^	2.04 ± 0.06 ^c^
**Coliforms Count (log CFU g^−1^)**			
0	2.10 ± 0.10	2.10 ± 0.10	2.10 ± 0.10
3	2.15 ± 0.06 ^a^	2.13 ± 0.02 ^a^	2.04 ± 0.04 ^b^
6	2.50 ± 0.20 ^a^	2.08 ± 0.04 ^b^	0.00 ± 0.00 ^c^
9	3.63 ± 0.15 ^a^	2.03 ± 0.06 ^b^	0.00 ± 0.00 ^c^
12	4.03 ± 0.25 ^a^	0.00 ± 0.00 ^b^	0.00 ± 0.00 ^b^
15	4.45 ± 0.26 ^a^	0.00 ± 0.00 ^b^	0.00 ± 0.00 ^b^

Every value is the average of three replicates ± SD. The different letters in the same row indicate significantly (*p* < 0.05) different values.

**Table 2:** The biological effects of the CFG-supplemented beef administered to albino rats during 28 days under standard conditions on the liver and kidney functions.

CFG/Diet (µg g^−1^)	CFG/Rat (mg/Kg)	ALT (U/l)	AST (U/l)	ALP (U/l)	Urea (mg/dl)	Creatinine (mg/dl)
0	0	24.8 ± 6.7	26.4 ± 1.81	138 ± 7.17	41.98 ± 1.76	0.5 ± 0.06
50	5	25.2 ± 6.01	27 ± 3.16	138.4 ± 5.95	42.58 ± 1.67	0.46 ± 0.02
100	10	25.6 ± 2.07	27.2 ± 1.92	139 ± 2.07	42.56 ± 0.73	0.48 ± 0.01
200	20	26.0 ± 3.11	27.5 ± 2.1	140 ± 3.17	43.45 ± 1.15	0.51 ± 0.03

There are no significant differences between the column values including the biochemical parameters data. Every value is the average of five replicates ± SD.
